# Selective Algicidal Action of Peptides against Harmful Algal Bloom Species

**DOI:** 10.1371/journal.pone.0026733

**Published:** 2011-10-26

**Authors:** Seong-Cheol Park, Jong-Kook Lee, Si Wouk Kim, Yoonkyung Park

**Affiliations:** 1 Research Center for Proteinaceous Materials (RCPM), Chosun University, Gwangju, Republic of Korea; 2 Department of Environmental Engineering, Chosun University, Gwangju, Republic of Korea; 3 Department of Biotechnology, Chosun University, Gwangju, Republic of Korea; US Dept. of Agriculture – Agricultural Research Service (USDA-ARS), United States of America

## Abstract

Recently, harmful algal bloom (HAB), also termed “red tide”, has been recognized as a serious problem in marine environments according to climate changes worldwide. Many novel materials or methods to prevent HAB have not yet been employed except for clay dispersion, in which can the resulting sedimentation on the seafloor can also cause alteration in marine ecology or secondary environmental pollution. In the current study, we investigated that antimicrobial peptide have a potential in controlling HAB without cytotoxicity to harmless marine organisms. Here, antimicrobial peptides are proposed as new algicidal compounds in combating HAB cells. HPA3 and HPA3NT3 peptides which exert potent antimicrobial activity via pore forming action in plasma membrane showed that HPA3NT3 reduced the motility of algal cells, disrupted their plasma membrane, and induced the efflux of intracellular components. Against raphidoflagellate such as *Heterosigma akashiwo*, *Chattonella sp.*, and *C. marina*, it displayed a rapid lysing action in cell membranes at 1∼4 µM within 2 min. Comparatively, its lysing effects occurred at 8 µM within 1 h in dinoflagellate such as *Cochlodium polykrikoides*, *Prorocentrum micans*, and *P. minimum*. Moreover, its lysing action induced the lysis of chloroplasts and loss of chlorophyll *a*. In the contrary, this peptide was not effective against *Skeletonema costatum*, harmless algal cell, even at 256 µM, moreover, it killed only *H. akashiwo* or *C. marina* in co-cultivation with *S. costatum*, indicating to its selective algicidal activity between harmful and harmless algal cells. The peptide was non-hemolytic against red blood cells of *Sebastes schlegeli*, the black rockfish, at 120 µM. HAB cells were quickly and selectively lysed following treatment of antimicrobial peptides without cytotoxicity to harmless marine organisms. Thus, the antibiotic peptides examined in our study appear to have much potential in effectively controlling HAB with minimal impact on marine ecology.

## Introduction

Harmful algal bloom (HAB), commonly called “red tide” and caused by massive and exceptional overgrowth of microalgae and cyanobacteria around the coasts, has increased globally with serious implications for the aquaculture industry and human health [1]. HAB not only leads to huge economic loss but also contributes to pollution of the coastal areas. This phenomenon often causes damage such as mass mortalities of fish, marine mammals, shellfish, and other oceanic life due to toxins and depletion of oxygen [2]. Furthermore, ingestion of seafood that has accumulated toxins produced by HAB results in illness and death in both humans and animals [2], [3]. Previous studies conducted in the Florida gulf coast have also found that exposure to aerosolized toxins from contaminated water leads to respiratory problems in humans [4], [5].

Many efforts have been made to prevent and mitigate the impact of HAB. Several reports suggest suppressing or killing harmful algal species using mechanical, biological, and chemical methods [6]–[10]. Despite several trials, a general method for controlling drastically increased blooming is limited due to differences in marine ecologies throughout the world. At times, secondary pollution of the marine environment is caused by imprudent treatments for preventing HABs. For example, clay or yellow loess was used to remove HAB organisms by sedimentation in Korea, Japan, and other countries; however, this method occasionally creates secondary effects on the bottom-dwelling organisms or ecological and environmental problems through dispersal of a large amount of clay to sea [11]–[13]. Likewise, although chemical compounds with algicidal activity have been isolated from various sources, it is possible to cause ecological changes as these reagents are not easily biodegraded and can accumulate in marine organisms over long periods similar to agricultural chemicals. Presently, alternative methods or materials are needed to minimize such problems.

It is well-known that several species of dinophyceae belonging to the genus *Prorocentrum* and *Cochlodinium* and raphidophyceae belonging to the genus *Chattonella* and *Heterosigma*, HAB cells used in present study, acutely produce toxins that poison shellfish and other marine organisms [14]–[16]. On the other hand, HPA3 (19-mer) and HPA3NT3 (15-mer) peptides, that are suggested to prevent HAB cells in here, are the modified antimicrobial peptides (AMPs) of the HP(2–20) peptide secreted from *Helicobacter pylori* [17]. They act against pathogenic bacteria and fungi through a pore-forming mechanism in plasma membranes [17].

AMPs have a potential for effectively managing HABs because they exert a selective algicidal action against toxic, rather than non-toxic, algal species. Moreover, compared to other chemical materials or microbiological treatments, HABs eliminate toxic algal organisms more quickly by bursting the cells. The aims of this study were (1) to determine algicidal activity of AMPs, (2) to prove the process of algal cell lysis after expose to an AMP under light microscopy, (3) to investigate selective targeting of toxic algal cells, and (4) to document the mode of algicidal action. Herein, we demonstrate the potent algicidal activity of an antimicrobial peptide (AMP) and its potential role as a novel material that selectively acts against these toxic algal species.

## Methods

### Materials

SYTOX Green was obtained from Molecular Probes (Eugene, OR) and 5(6)-carboxytetramethylrhodamine N-succinimidyl ester (TAMRA) were purchased from Sigma-Aldrich (St. Louis, MO). 9-fluorenylmethoxycarbonyl (Fmoc) amino acids were from CEM Co. (Matthews, NC). All other reagents were of analytical grade.

### Peptide synthesis

Peptides were synthesized by Fmoc solid-phase methods on Rink amide 4-methyl benzhydrylamine resin (Novabiochem; 0.55 mmol/g) by using a Liberty microwave peptide synthesizer (CEM Co.). The protocols for peptide cleavage and purification used for this study were previously described [17].

### Algal cultures

*Prorocentrum minimum* (D-120), *Prorocentrum micans* (D-077), *Heterosigma akashiwo* (RA-020, RA-018), *Chattonella sp*. (RA-005), and *Skelectonema costatum* (B-796) were provided by the Korea Marine Microalgae Culture Center, Busan, Republic of Korea. *Cochlodinium polykrikoides*, *H. akashiwo*, and *Chattonella marina* were collected for this study from the coastal area of the South Sea in Korea. All but one species were grown and maintained in an f/2 medium [18] at 20°C with 14/10 h light/dark illumination cycles under cool white fluorescent light (120 µmol photons m^−2^s^−1^). *S. costatum* was grown under the same conditions except that this organism was cultured with a light density of 30 µmol photons m^−2^s^−1^.

### Algicidal activity

Algal cultures grown at mid-exponential phase were introduced to a 24-well tissue culture plate at a concentration of 2∼4×10^4^ cells/mL, and two-fold serial dilutions of each peptide with f/2 medium were added in a range from 0.25 to 256 µM. After a 1 h (for raphidophyceae), 4 h (for dinophyceae), or 72 h (for *S. costatum*) incubation, the numbers of surviving cells were counted in a hemacytometer with a Sedgwick–Rafter counting chamber under an inverted microscopy. Inhibitory concentrations (ICs) of peptides were defined by counting the number of burst cells in which cell envelopes or membranes were completely disrupted, but not non-swimming cells. Under the same conditions, peptides were added at IC_90_ to *C. marina* cultures and the time-dependent algicidal action of the peptides was recorded under inverted microscopy with a DP71 camera (Olympus, Tokyo, Japan).

Pre-cultured *H. akashiwo* and *P. minimum* cells were adjusted to a concentration of 2.1×10^4^ cells/mL and *S. costatum* cell cultures were adjusted to 4.2×10^4^ cells/mL. After mixing cultures of *H. akashiwo* with *S. costatum* or *P. minimum* with *S. costatum*, 2 or 8 µM of HPA3NT3 were added and cells were counted at indicated times in a hemacytometer with a Sedgwick–Rafter counting chamber.

### Confocal laser scanning microscopy (CLSM)

The cellular distribution of peptides was examined by using TAMRA-labeled peptides and CLSM. Algal cells were incubated with TAMRA-HPA3 or -HPA3NT3 at IC_50_ for 5 min (*H akashiwo* and *C. marina*) or 1 h (*P. minimum*), after which the cells were washed three times with f/2 medium by centrifugation (×1000 g, 5 min) and fixed with 2% (v/v) glutaraldehyde. Localization of TAMRA-labeled peptides was then observed using an inverted LSM510 laser-scanning microscope (Carl Zeiss, Gőttingen, Germany). To detect TAMRA-labeled peptides, 543-nm light from a helium neon laser was directed at a DIC/543 beam splitter. Images were then recorded digitally in a 512×512 pixel format in serial sections from the top to bottom of the algal cells.

### Assay for chlorophyll *a*

To measure chlorophyll *a* concentrations, *H. akashiwo* cells incubated in the absence or presence of the peptides were harvested by centrifugation (at 3,000×g for 10 min) at the indicated times. The cell pellets were resuspended and extracted in 90% acetone for 24 h at 4°C. The samples were then centrifuged at 10,000×g for 10 min to remove cell debris and chlorophyll a concentrations were determined as described by in Jeffrey and Humphrey [18].

### SYTOX green uptake

Algal cells were grown to mid-exponential phase in the above culture condition and adjusted to 4×10^4^ cell/mL in f/2 media, after which they were incubated with 0.5 µM SYTOX Green for 15 min in the dark. After addition of the peptides at the indicated concentrations, an increase in fluorescence from the binding of the cationic dye to intracellular DNA was monitored over time. The excitation and emission wavelengths were 485 nm and 520 nm, respectively. All fluorescence values were plotted by base fluorescence that was obtained in algal cells without peptide.

### Hemolysis

Fresh fish blood was collected from *Sebastes schlegeli* and immediately injected into heparin blood collection tubes (BD Vacutainer, Franklin Lakes, NJ). After gently mixing, red blood cells (RBCs) were centrifuged at 800×g and washed with PBS until the supernatant was clear. The RBCs (8% (v/v) of the final concentration) were added to 2-fold serially dilutions of peptide with PBS. After incubation with mild agitation for 1 h at 37°C, the samples were then centrifuged at 800×g for 10 min and absorbance of the supernatant was then measured at 414 nm. Complete (100%) or no hemolysis was defined as the absorbance of the RBCs containing 1% Triton X-100 or PBS alone, respectively. Each measurement was made in triplicate, and percentage hemolysis was calculated using Equation 1:
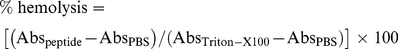
(1)


### Time-dependent elimination of red tide by peptide

*H. akashiwo* cells grown at mid-exponential phase were transferred to a hexahedron cell (12×12×45 mm) at a density of 1×10^8^ cells/m: and then incubated with 40 µg/mL of HPA3NT3 peptide. Immediately after adding the peptide, the reduction in viable cell numbers was continuously recorded for 20 min by using a digital video recorder. The individual images were extracted at 1 min intervals by video imaging software.

## Results

### AMPs have potent algicidal activity against harmful algal species

As shown in Table S1, they had a higher hydrophobicity and hydrophobic moment than parent peptide, HP (2–20), indicating that they favor interaction with cellular membranes. The anti-algal activity of HPA3 and HPA3NT3 peptides was evaluated by the observation of decreased motility and bursting of harmful algal species, *Cochlodinium polykrikoides*, *Prorocentrum minimum* and *micans* (dinophyceae or dinoflagellate), *Chattonella marina* and *sp.*, and *Heterosigma akashiwo* (raphidophyceae or raphidoflagellate) under light microscopy (Table 1). HP (2–20) led to weak growth-inhibition in almost algal cells, while HPA3 and HPA3NT3, having additional cationicity and hydrophobicity, were more potent. HPA3NT3 had better algicidal action against all harmful algal species compared to the other peptides, at concentrations ranging from 1 to 8 µM. This peptide was particularly more active against raphidoflagellate than dinoflagellate. Generally, swimming movements of the algal cells were inhibited by treatments with the peptides at 1/4 concentrations of IC_90_, and lysis of occurred at concentrations over IC_50_.

**Table 1:** The algicidal activity of the peptides against various marine algal strains.

Strains	Inhibitory concentration (µM)					
	HP(2–20)		HPA3		HPA3NT3	
	IC_50_	IC_90_	IC_50_	IC_90_	IC_50_	IC_90_
**Dinophyceae**						
*Cochlodinium polykrikoides* *	64	128	4	8	4	8
*Prorocentrum minimum* D-120	128	>256	16	32	4	8
*Prorocentrum micans* D-077	128	>256	16	32	4	8
**Raphidophyceae**						
*Heterosigma akashiwo* RA-020	32	128	2	2–4	0.5	1
*Heterosigma akashiwo* RA-018	64	128	2	2–4	0.5	1
*Heterosigma akashiwo* *	64	128	2	4	0.5	1
*Chattonella sp.* RA-005	64	128	2	4	1	2
*Chattonella mirina* *	32	64	4	8	2	4
**Bacillariophyceae**						
*Skeletonema costatum* B-796	256	>256	128	>256	>256	>256

*Algal strains for this study were isolated from the South Sea of Korea.

**Figure 1:**
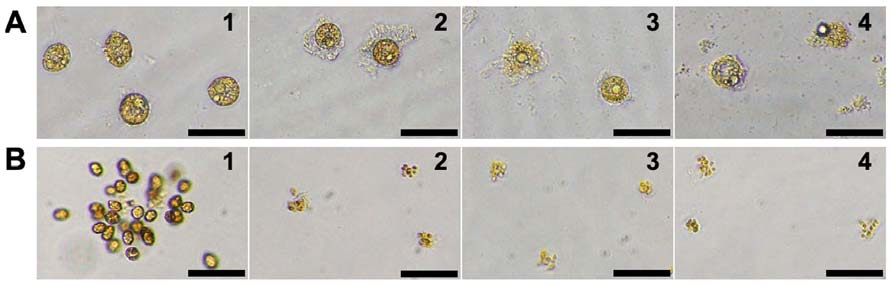
Morphological changes of rapidoflagellates in the presence of peptides. *C. marina* (A) and *H. akashiwo* RA-018 (B) cells incubated without (1) or with HP(2–20) (2), HPA3 (3), and HPA3NT3 (4) peptides each at IC_90_ for 2 min. Bar: 100 µm for all panels.

**Figure 2:**
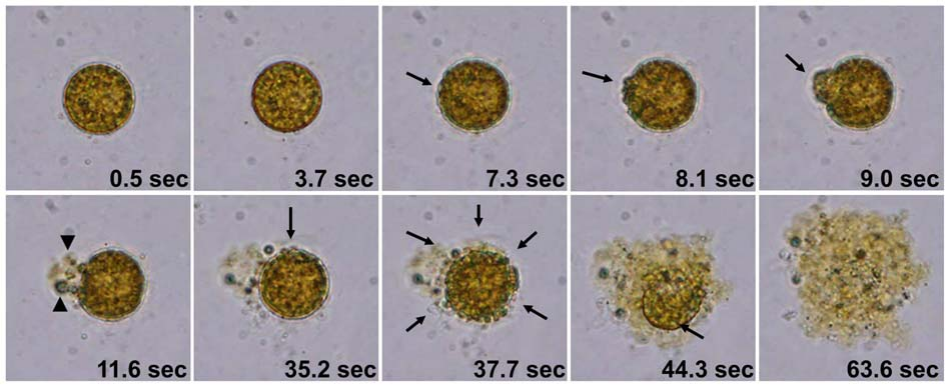
Time-dependent changes of *C. marina* cells in the presence of HPA3NT3. After 4 µM of peptide of was introduced to algal cells, cell morphology was digitally recorded by video. The images were extracted from video files at the indicated times. Arrows and arrowheads indicate the damaged regions of the plasma membrane and the released chloroplasts, respectively.

Upon exposure to HPA3 and HPA3NT3, *C. marina* and *H. akashiwo* cells swelled, their plasma membranes were disrupted, and cytoplasmic organelles and materials were released. Ultimately, cell lysis was complete within 2 min (Fig. 1 and Movie S1). This short period required for cell destruction suggested that electrostatic force of peptides led to rapid binding on algal cell membranes although membrane lipid composition is diverse according to algal species. Fig. 2 and Movie S2 show the time-dependent lysis of *C. marina* cell in the presence of HPA3NT2. With the addition of peptide, motility of the cell stopped, cells became rounded (0.5 sec) by inducing permeability in cell membrane, all within a very short period. At 7.3 sec, the integrity of plasma membrane was changed. Continuously, a small swelling on the cells expanded (arrow at 8.1 and 9.0 sec), burst, and intracellular organelles were released (arrowheads at 11.6 sec) through one part of the membrane that was attacked by many peptides. Interestingly, lysis of chloroplast membranes resulted in loss of chloroplast coloring. Images taken at 37.7 sec showed that the lysis of the plasma membrane and release of chloroplast membranes were ongoing in several parts of the cell. A layer of cell membrane disappeared and chloroplasts were almost destroyed at 63.6 sec.

**Figure 3:**
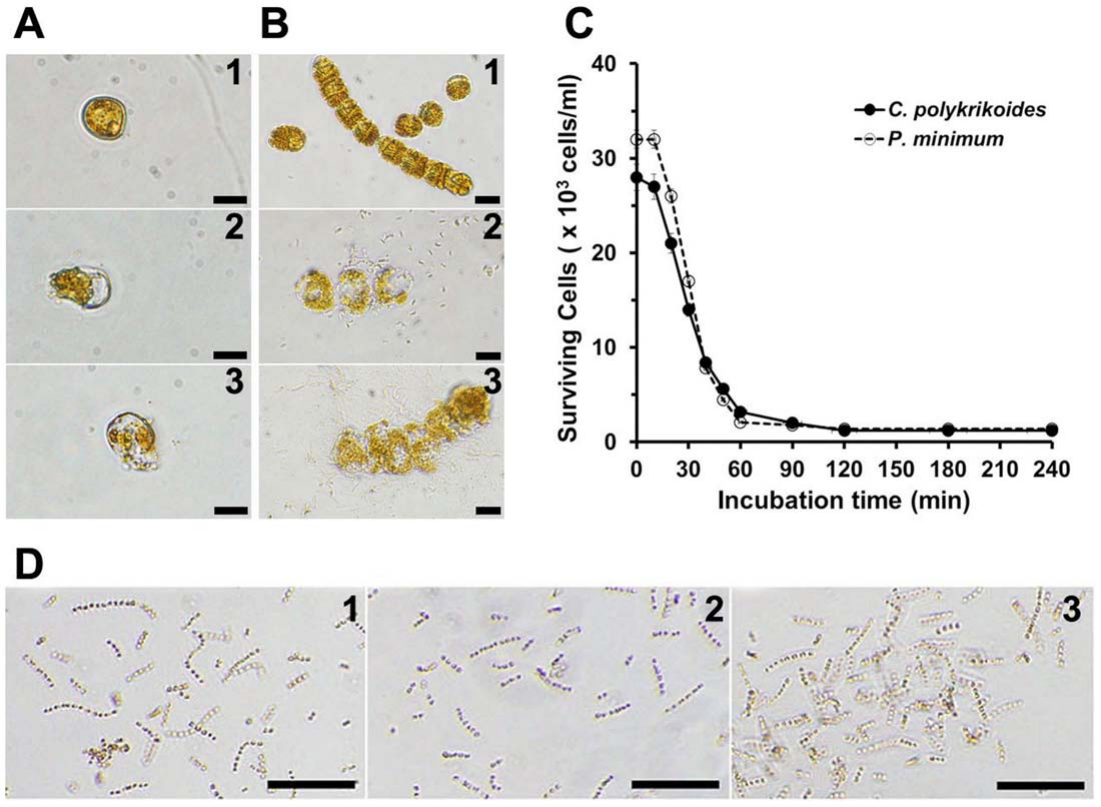
Algicidal effects of peptides against dinoflagellates and diatoms. (A and B) *P. minimum* (A) and *C. polykrikoides* (B) cells without (1) or with HPA3 (2) and HPA3NT3 (3) each at each IC_90_. (C) Density of surviving cells at the indicated times. (D) *S. costaum* cells incubated without (1) or with 128 µM HPA3 (2) and 256 µM HPA3NT3 (3) for 3 days at 20°C. Bars: 50 µm for panels A and B and 100 µm for panel D.

As shown in Fig. 3A and B, the mode of action of HPA3 and HPA3NT3 on *C. polykrikoides* and *P. minimum* was similar to that on raphidoflagellate, but complete lysis of dinoflagellate required long-term exposure to the peptides (1 h) compared to raphidoflagellate (2 min). Long chains of *C. polykrikoides* were broken and motility of *P. minimum* was significantly decreased during the first 10 min after introducing of peptides, but changes in morphology of individual cells was remarkable. Subsequently, non-motile cells expanded, cell envelopes and membranes were destroyed, and efflux of intracellular materials followed within 1 h (Fig. 3A–C). Differences in the kinetics of initial cell interaction and complete lysis between dinoflagellate and raphidoflagellate were dependent on the absence or presence of cell envelope and the number of outer membranes of the chloroplasts.

On the other hand, although the growth of *S. costatum*, a harmless diatom, was inhibited at 256 µM of HPA3, half of cells were survival (Fig. 3D–2). However, a long-term exposure to 256 µM HPA3NT3 did not lead to any changes in morphology or survival of *S. costatum* cells (Fig. 3D–3).

### Selective algicidal action of HPA3NT3

**Figure 4:**
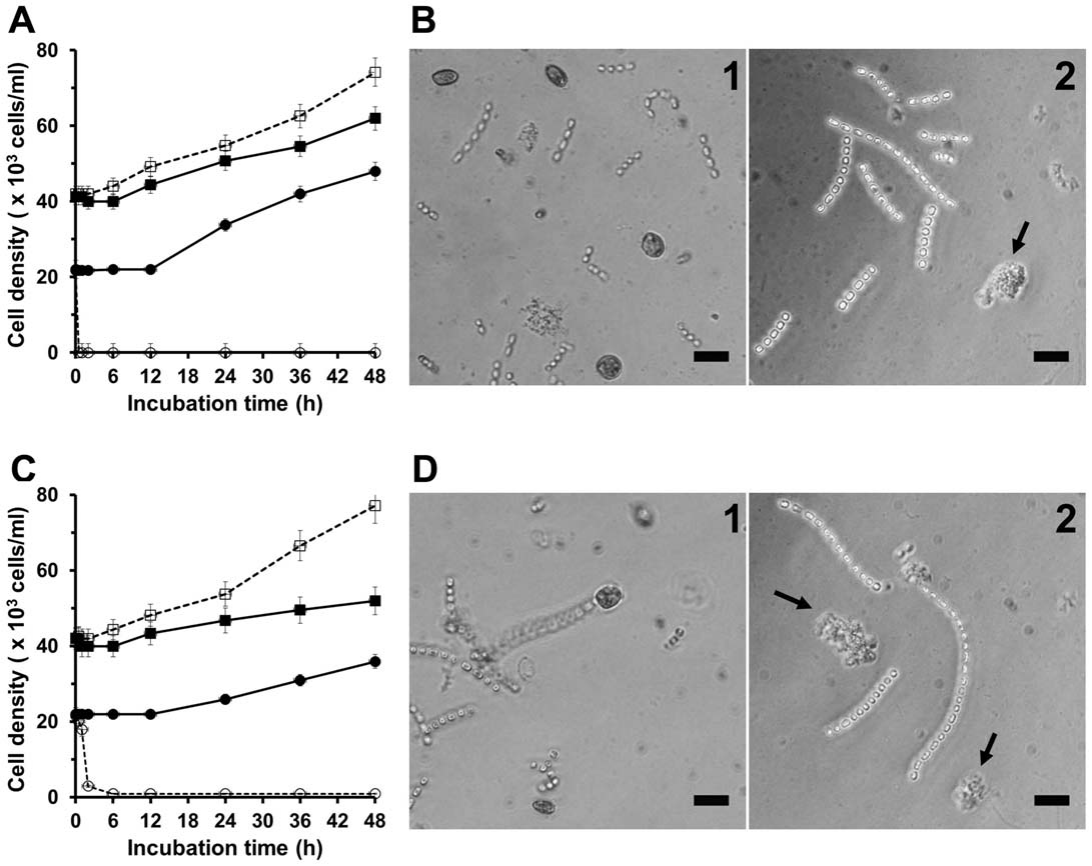
The selective algicidal action of HPA3NT3 in co-cultivations of harmful and harmless algal cells. (A) The changes of cell density in co-cultures of *S. costatum* (square) and *H. akashiwo* (circle) in the absence (filled and 1) or presence (open and 2) of 2 µM HPA3NT3. (B) Images of surviving cells after 6 h of incubation with and without 2 µM HPA3NT2. (C) The changes of cell density in co-cultures of *S. costatum* (square) and *P. minimum* (triangle) in the absence (filled and 1) or presence (open and 2) of 8 µM HPA3NT3. (D) Images of surviving cells after 24 h of incubation with and without 8 µM HPA3NT2. Bar: 50 µm for panels B and D.

To investigate selective algicidal action of HPA3NT3, harmful (*H. akashiwo* or *P. minimum*) and harmless (*S. costatum*) algal cells were co-cultured in the presence of the peptide. After pre-culturing both types of algal cells, *S. costatum* cultures (4.2×10^4^ cells/mL) were mixed with *H. akashiwo* or *P. minimum* cultures (2.1×10^4^ cells/mL) in different cell densities because *S. costatum* can be preyed upon by HAB [19], [20]. HPA3NT3 was introduced to combinations of co-cultured cells at concentrations of 2 µM or 8 µM (IC_90_ against *H. akashiwo* and *P. minimum*, respectively). Survival of the algal cells was monitored over time for 48 hr (Fig. 4A and C) and the cells were counted. In control experiment without peptide, the growth patterns of the two co-cultures in the absence of peptide were shown that growth of *S. costatum* with *P. minimum* was little inhibited compared to co-culture of *S. costatum* and *H. akashiwo* (Fig. 4A and C) because *S. costatum* was purposely fed to *P. minimum*. While addition of HPA3NT3 resulted in *H. akashiwo* or *P. minimum* cells lysis and aggregation within 10 min (Fig. 4B–2- arrow) or 1 h (Fig. 4D–2- arrow), respectively, cell density of *S. costatum* was significantly increased for 24 h although its growth rate was slightly inhibited for 2 (Fig. 3A) or 6 h (Fig. 3C) following peptide exposure. In addition, presence of peptide led to enhanced *S. costatum* growth, indicating that selective killing and rapid lysis action of HPA3NT3 against only harmful algal cells played an important role in the propagation of *S. costatum*.

### Cellular targets of HPA3 and HPA3NT3 algicidal action

**Figure 5:**
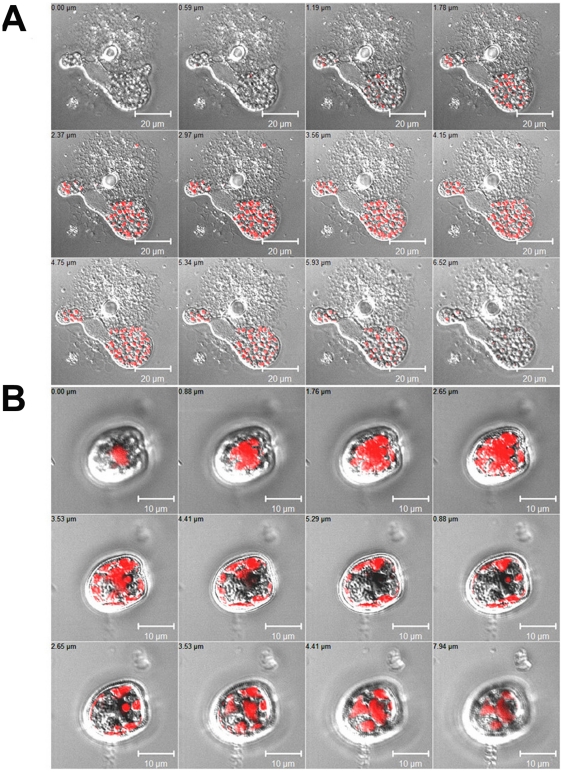
Localization of TAMRA-HPA3NT3 in serial sections. (A) *C. marina* cell treated with 2 µM of TAMRA-HPA3NT3 for 2 min. (B) *P. minimum* cell treated with 4 µM of TAMRA-HPA3NT3 for 30 min. The images were automatically recorded on 12 planes from the top to bottom of the cell. Numbers in left top of each panel represent the distance of focus from the top of the cell.

To identify cellular regions of peptide action in harmful algal cells, the N-termini of HPA3 and HPA3NT3 were labeled with carboxytetramethylrhodamine (TAMRA) to produce the peptides TAMRA-HPA3 and TAMRA-HPA3NT3. Prior to this experiment, we examined whether labeling with this dye has any effect on the peptide algicidal activity, and found that the peptides' biological functions and abilities were not limited. Algal cells incubated with TAMRA-labeled peptides at IC_50_ were observed under confocal laser scanning microscopy (CLSM). In order to minimize a misjudgement of planes due to the large size and dimension of algal cells, images were serially recorded on 12 planes from the top to bottom of the cells. The localization of TAMRA-HPA3 in the cell surfaces appeared as an intensive red pigmentation on the plasma membrane and chloroplasts of *H. akashiwo* and *C. marina* (Fig. S1). Likewise, *P. minimum* with thick envelopes of cellulose was also targeted (Fig. S1), although how the peptides passed across these envelopes was unclear. As shown in Fig. 5, chloroplast membranes were a main target of TAMRA-HPA3NT3. In order to investigate other intracellular targets, *C. marina* cells in which lysis was ongoing were examined (Fig. 5B). Fluorescence was more concentrated in chloroplasts than in the plasma membrane and released cytoplasmic materials. This indicated that HPA3NT3 possesses significant affinity for the chloroplast envelope and thylakoid membranes composed of phosphatidylglycerol and sulfoquinovosyldiacylglycerol [21], [22]. Cationic peptide binds to negatively charged lipids through electrostatic interactions.

### Mode of action of HPA3NT3

**Figure 6:**
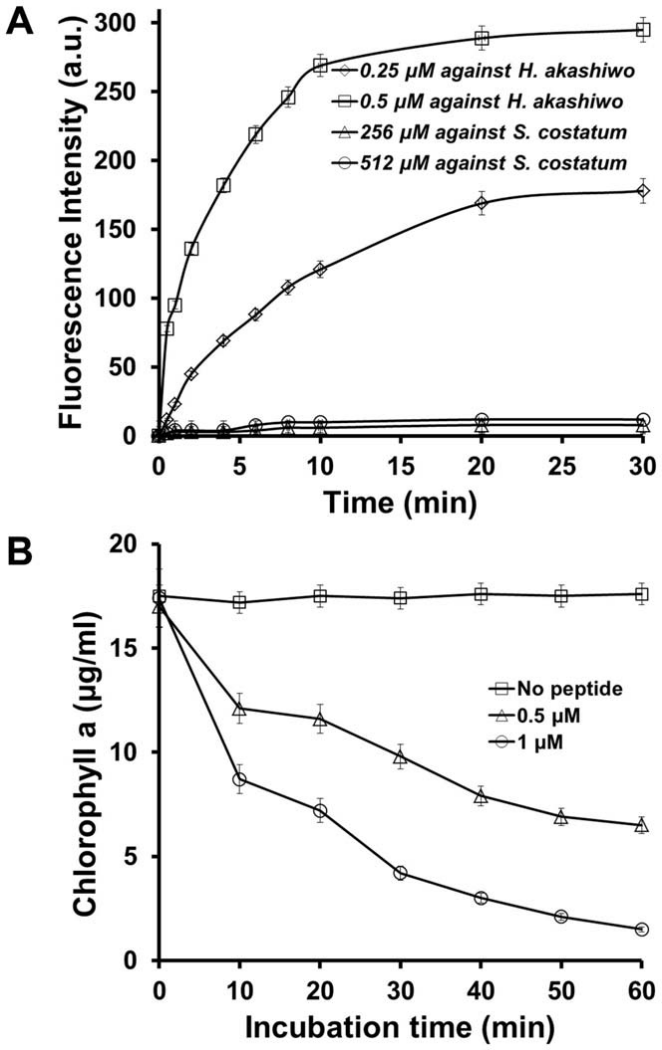
Mode of action of HPA3NT3 in algal cells. (A) Uptake of SYTOX green in *H. akashiwo* and *S. costatum* cells treated with HPA3NT3. (B) Concentrations of chlorophyll a in cell treated with HPA3NT3.

To determine how HPA3NT3 kills HAB cells, we investigated the peptide-induced damage in two regions, the plasma membrane and chloroplast. First, uptake of SYTOX green, a vital dye which does not penetrate the membrane of live cells by itself, was used to analyse *H. akashiwo* and *S. costatum* cells treated with HPA3NT3. After pre-incubating algal cells with this dye, HPA3NT3 was added at 1/2 and 1×IC_50_ that resulted in only loss of cell motility (Fig. 6A). In this assay, the reason of peptide treatment at not IC_90_ but IC_50_ value was that nucleic acids released by complete lysis of algal cells at IC_90_ can bind with non-penetrating or free dyes. In *H. akashiwo* cells, fluorescence emitted upon dye binding to cytoplasmic nucleic acids was increased in a concentration- and time-dependent manner, indicating that HPA3NT3 directly acts on plasma membranes leading to an influx of dye. Additionally, maximal fluorescence intensity was recorded at 10 min in the presence of 0.5 µM HPA3NT3. On the contrary, HPA3NT3 did not induce permeability of the plasma membrane in *S. costatum*, a harmless diatom, as evidenced by no change in fluorescence over 30 min in the presence of the peptide; further incubation with the peptide for 2 h did not induce any change in fluorescence (data not shown).

We next investigated the effect of HPA3NT3 peptide on chloroplasts because these organelles leaked out from *H. akashiwo* cells upon damage to the plasma membranes. Chlorophyll *a* concentrations in the control cells not exposed to the peptide were maintained for almost 60 min, but the concentrations in cells treated with 0.5 and 1 µM HPA3NT3 were significantly reduced in a time-dependent manner (Fig. 6B). These data indicate that the peptide disrupts both the envelope and thylakoid membranes of chloroplasts, corresponding to the rapid loss of green coloring observed in the cells.

### Cytotoxicity of peptides against fish red blood cells (fRBCs)

**Figure 7:**
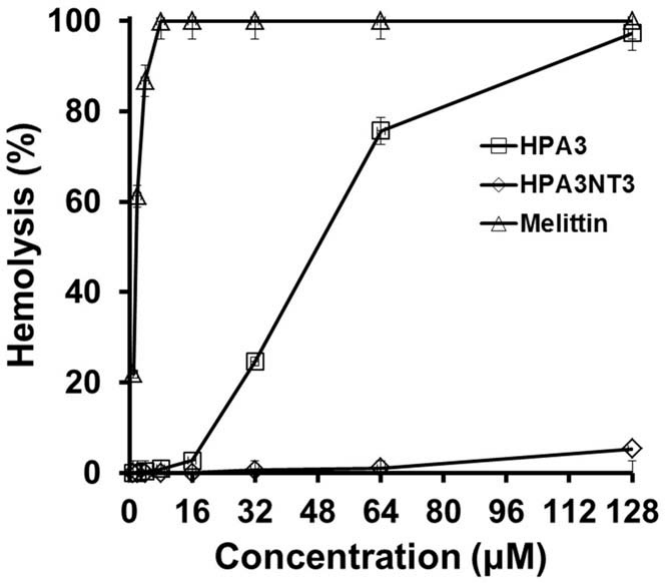
Hemolytic effects of peptides. Peptides with indicated concentrations incubated for 1 h with red blood cells collected from *Sebastes schlegeli*, and the released hemoglobin was then calculated.

We collected RBCs from rockfish, *Sebastes schlegeli*, in order to examine cytotoxic effects of the peptides on fish cell. Melittin, a cytotoxic peptide which was used as a negative control, caused 100% hemolysis at 8 µM, but HPA3 and HPA3NT3 induced 75.6% and 1.0% hemolysis, respectively, at 64 µM (Fig. 7). Treatment with 128 µM HPA3NT3, 128 times IC_90_ for *H. akashiwo* cells, resulted in 5.3% hemolysis. These data suggested that HPA3NT3, but not HPA3, may be used as an algicidal agent without being cytotoxic to other marine organisms.

### *In vitro* clearance of red tide by addition of HPA3NT3

**Figure 8:**
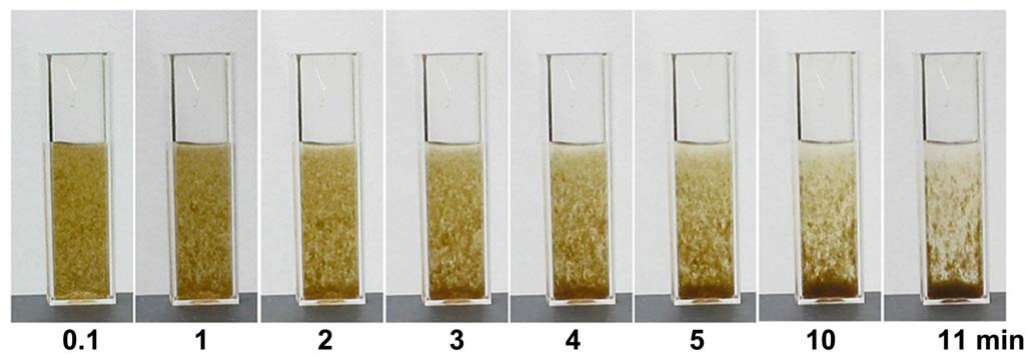
*In vitro* clearance of red tide. 40 µg/mL of HPA3NT3 was mixed with *H. akashiwo* of 1×10^8^ cells/mL, and sedimentation of algal cells or clearance of turbid seawater continuously recorded.

To further investigate the effectiveness of HPA3NT3 in controlling HAB and the associated high densities of harmful algal cells, we scaled up an algicidal assay for this peptide. As shown in Fig. 8, 1×10^8^ cells/mL of *H. akashiwo* were incubated with 40 µg/mL of HPA3NT3, and the reduction of the algal bloom was continuously recorded for 20 min. The algal bloom immediately reacted to the addition of peptide, and debris from lysed cells began to precipitate at 1 min. Continuous sedimentation was almost completed at 11 min and the extremely turbid media was clear (Fig. 8). This rapid clearance corresponded to the rapid peptide-induced plasma and chloroplast membrane lysis in harmful algal cells.

## Discussion

During the recent decades, problems with HABs have significantly increased due to factors such as extensive costal eutrophication and global climate change [23]–[25]. Suggested methods for controlling HABs are limited to a few strategies such as the use of algicidal viruses [26], [27], predators of toxic algal species [19], [28], algicidal agents produced from bacteria [8], [9], [29], UV irradiation [30], [31], and clay sedimentation [6], [12]. However, the only used method is flocculation of harmful algae through clay dispersal [6], [7], [12], but this method is associated with secondary contamination and changes in marine environments and ecologies when the clay was highly dispersed [11], [13]. Presently, new materials or methods that are able to substitute for the above management strategies are urgently required.

The study on the algicidal activity of antibiotic peptides against harmful algal species is rare, although one peptide with two standard and three unusual amino acids containing hydroxyl groups has been reported [32]. In our study, we demonstrated that antimicrobial peptides may be useful in the prevention of HABs due to several characteristics: (1) selective algicidal action, (2) membranolytic action, (3) non- or low cytotoxic, and (4) standard amino acid composition.

### Potent and selective algicidal activity of HPA3 and HPA3NT3 antimicrobial peptides

We hypothesized that membranolytic action of HPA3 and HPA3NT3 would lead to algicidal activity because eukaryotic phytoplankton cells are usually surrounded by phospholipid and glycolipid bilayers although the lipid composition varies according to species. In this study, we showed that both HPA3 and HPA3NT3 have a potent algicidal activity against two types of harmful algal cells through lysing cell membranes and chloroplasts. Other studies reported that some fatty acids with anti-algal activity cause a leakage of intracellular K^+^ resulting from damage to the plasma membrane in phytoplankton and cyanobacteria, and dissociated phycobilins from thylakoids [33]–[35]. Sokolov *et al*. [36] suggested that fatty acids are able to cause membrane permeability through the formation of ion channels in the phospholipid bilayers, thereby altering the membrane structure.

The present study revealed that algicidal action of both peptides were more rapid and effective against raphidoflagellate such as *H. akashiwo*, *C. marina*, and *C. sp*. than dinoflagellate like *C. polykrikoides*, *P. minimum*, and *P. micans.* Generally, cell envelopes of raphidoflagellate are mainly composed of glycocalyx and plasma membranes, but dinoflagellate used in this study possesses an “armored” type of cell wall in which the plasma membrane is typically covered by thick plates of cellulose [37]–[40]. It was known that morphologies of raphidoflagellate are easily changed by chemical and physical treatment [39], therefore it was not surprising to observe that the efflux of intracellular components from algal cells was quickly induced by the peptides. Generally AMPs with cationicity act to membrane and have membrane permeating activity, therefore, they can be easily bind to plasma membrane of raphidoflagellate and can disrupt through electrostatic interaction and amphipathic character. This effect required more time in dinoflagellate because the peptides are difficult to reach the plasma membrane across cellulose layers of their cell wall and the breakdown of this layer was not easy. Moreover, as shown in Fig. 2 and Fig. 6, the peptides interacted with and lysed the chloroplasts and other cell organelles in harmful algal cells. Raphidoflagellate chloroplasts are composed of two membrane layers while dinoflagellate contains two of chloroplast envelope plus postulated plasmalemma [41]–[43]. This may explain the differences in the kinetics of peptide action against raphidoflagellate and dinoflagellate.

*S. costatum* is a ubiquitous and relatively harmless diatom (bacillariophyceae) which provides to coastal fisheries as a food source due to fast growing rate and plays a role of marine re-mineralization [44], [45] although it is often forms spring blooms in China [46]. The population and growth of *S. costatum* was not affected by the HPA3NT3 peptide, even at high concentration. In addition, co-culture experiments with harmful and harmless algal cells showed that only harmful algal cells were killed by the peptide without any impact on the harmless algal cell (Fig. 4). Generally, an organic casing of the diatom cell wall is coated by siliceous frustule which is forms a joint or gasket through callose, a structural β-1,3-linked glucan [47], [48]. Perhaps, this siliceous coat will interrupt the peptides' attack by providing a protective barrier that prevents the peptide from reaching the plasma membrane. This may contribute to the selective algicidal activity of the HPA3NT3 peptide.

### Plausible mode of action of HPA3 and HPA3NT3

As shown in the microscopic observation, both peptides disrupted the plasma and chloroplast membranes of harmful algal cells. TAMRA-HPA3NT3 localized to the chloroplast membranes more than the plasma membranes of *H. akashiwo* and *P. minimum* (Fig. 5A). In particular, *C. marina* cells undergoing lysis of the plasma membrane showed concentrated fluorescence in the chloroplasts (Fig. 5B). Results from experiments measuring the influx of SYTOX Green dye, which flowed into the cytoplasm and led to increased fluorescence upon binding to nucleic acids when the plasma membrane was damaged, provided insight into the membrane-permeable action of the peptides. HPA3NT3 induced a significant increase in fluorescence in *H. akashiwo* cells for 10 min at concentrations below MIC_90_, meaning that the peptide damaged the plasma membrane. In addition, concentrations of chlorophyll a were decreased by treatment with HPA3NT3. Since both peptides are amphipathic compounds containing hydrophobic and hydrophilic moieties, they can easily adhere on the plasma membrane and insert themselves into the lipid bilayer. Therefore, we suggest that the entrance of peptides through the damaged plasma membrane allows targeting of the chloroplast membranes. Moreover, chloroplast membranes contain sulfoquinovosyldiacylglycerol and phosphatidylglycerol [41], negatively charged lipids, to which cationic peptides are able to bind through electrostatic interactions. On the other hand, plasma membranes of dinoflagellate used in this study are covered by cellulose plates; however, the peptides were able to kill these cells within 1–2 hr. Although the binding affinity of the peptides for cellulose was weak [17], they slowly pass through the polysaccharide layer and disrupt plasma and chloroplast membranes. In contrast to raphidoflagellate, *S. costatum* did not effectively uptake SYTOX Green in the presence of 512 µM of HPA3NT3. We suggest that the interaction of peptide with the interior plasma membrane may be protected by the siliceous cover.

### Cytotoxicity of peptides and *in vitro* clearing of red tide

The cytotoxicity of anti-algal compounds must be considered before using these reagents in marine environments. The present study showed that 128 µM of HPA3 and HPA3NT3 caused 97.3% and 5.3% hemolysis, respectively, in fresh red blood cells (RBCs) collected from *Sebastes schlegeli*, a black rockfish, indicating that HPA3NT3 peptide is less cytotoxic. Moreover, it was previously reported that HPA3NT3 possesses low hemolytic and cytotoxic activity against human RBCs and HaCaT cell [17]. The membranolytic action of HPA3NT3 prefers negatively charged phospholipids (such as PG, cardiolipin, and phosphatidylserine) over zwitterionic phospholipids (such as phosphatidylethanolamine and phosphatidylcholine) [17]. This strong action for negatively charged phospholipids particularly contributes to the selective lysis of harmful algal cells. Although HPA3NT3 cytotoxicity should be studied in other organisms, we propose that this peptide is a safe algicidal compound at least in fish. *In vitro* clearance experiments showed that dispersed HPA3NT3 removed algal bloom from top to bottom with 11 min through rapid sedimentation of lysed algal cells (Fig. 8). This experiment performed in high density of algal cells showed that the peptide can quickly control algal-blooming in limited areas.

As the peptides used in this study were composed of common amino acids, there are a number of reasonable advantages for using these for controlling HABs. First, these peptides can be naturally biodegraded in marine environments. Biodegradable property is one of important factors selecting algicidal materials for use in the ocean because algicidal chemicals are frequently noxious to marine organisms or human, and like agricultural chemicals, can accumulate in humans through the food chain. Therefore, these peptides, which are easily biodegraded in marine ecosystems, are comparatively safe. Another potential of them is that their sequences are easily able to be converted into genetic resource and to produce in a great quantity because they are ribosomally synthesized. Recent studies proposed that HABs can be controlled by parasites of harmful algal species [49] or single-stranded RNA viruses [26], [27]. Furthermore, more active and safe peptides could be developed by substitution of amino acids based upon the amino acid sequence of HPA3NT3 .

In practical terms, use of antimicrobial peptides in preventing HABs will be limited due to the high cost of chemical synthesis. This problem could be solved by processes such as mass production in microbes or plants, and enhancement of algicidal by gene-transfer into specific parasites or RNA viruses for harmful algal cells as these peptides are composed entirely of standard amino acids. Although we are only beginning to realize the potential of antimicrobial peptides in this field, further investigation will result in the discovery of practical applications for these peptides.

## Supporting Information

Figure S1 **Localization of TAMRA-HPA3 in algal cells.** (A) *H. akashiwo* cell treated with 2 µM of TAMRA-HPA3 for 2 min. (B) *C. marina* cell treated with 4 µM of TAMRA-HPA3 for 2 min. (C) *P. minimum* cell treated with 16 µM of TAMRA-HPA3 for 30 min. All images were recorded with a intermediated focus between the top and bottom of cells.
(TIF)

Table S1 **Sequence, molecular mass, mean hydrophobicity (** ***H*** **), and mean relative hydrophobic moment (** *µ* **_H_) of the peptides used in this study.**
(PDF)

Movie S1 **The morphological changes of** ***C. marina*** **with 4 µM of HPA3 were digitally recorded by video under microscopy.**
(MPG)

Movie S2 **After 4 µM of HPA3NT3 of was introduced to** ***C. marina*** **, cell morphology was digitally recorded by video under microscopy. Snapshots of** **Figure 2** **were extracted from this movie file.**
(MPG)
