# Supplementary figures and images for: Selective Algicidal Action of Peptides against Harmful Algal Bloom Species
Source: PLoS One. 2011 Oct 26;6(10):e26733. doi: 10.1371/journal.pone.0026733 (PMC3202551; doi:10.1371/journal.pone.0026733)

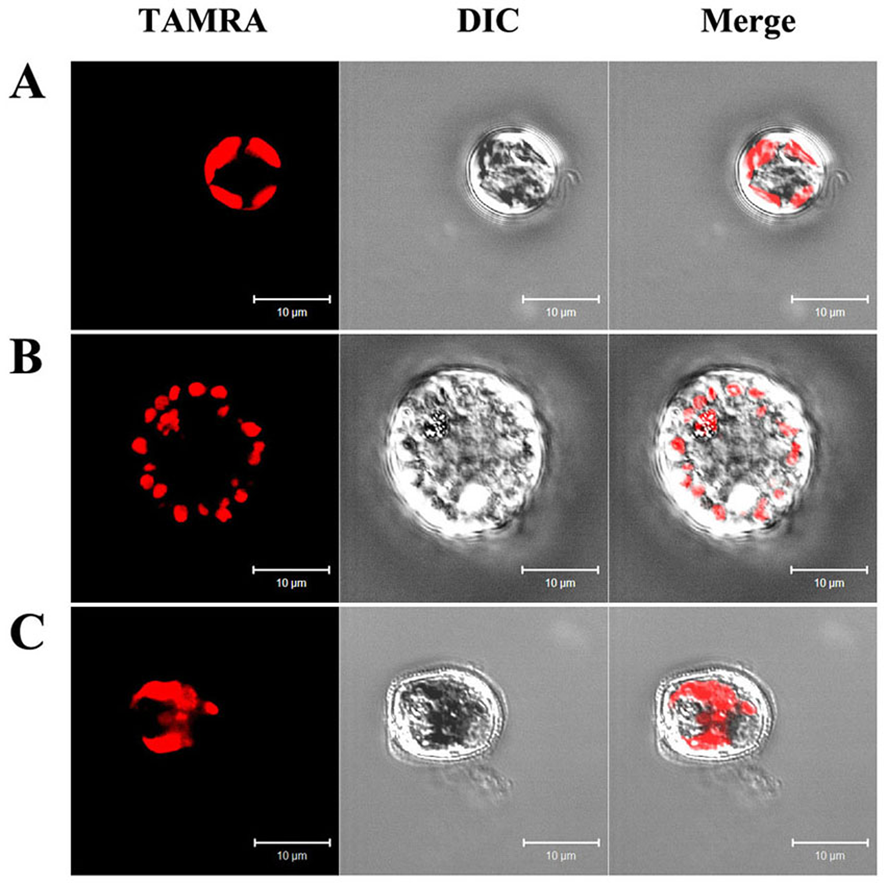

Supplement: Figure S1 — Localization of TAMRA-HPA3 in algal cells. (A) H. akashiwo cell treated with 2 µM of TAMRA-HPA3 for 2 min. (B) C. marina cell treated with 4 µM of TAMRA-HPA3 for 2 min. (C) P. minimum cell treated with 16 µM of TAMRA-HPA3 for 30 min. All images were recorded with a intermediated focus between the top and bottom of cells. (TIF) [file pone.0026733.s001.tif]
