# Supplementary material for: Selective Algicidal Action of Peptides against Harmful Algal Bloom Species
Source: PLoS One. 2011 Oct 26;6(10):e26733. doi: 10.1371/journal.pone.0026733 (PMC3202551; doi:10.1371/journal.pone.0026733)
Supplement: Table S1 — Sequence, molecular mass, mean hydrophobicity ( H ), and mean relative hydrophobic moment ( µ H) of the peptides used in this study. (PDF) [file pone.0026733.s002.pdf]

Table S1

Table S1. Sequence, molecular mass, mean hydrophobicity (*H*), and mean relative hydrophobic moment (μ_H) of the peptides used in this study.

Peptide	Sequence	Observed molecular mass	*H*	μ_H
HP(2-20)	AKKVFKRLEKLFSKIQNDK-NH_2	2320.0	-2.79	0.63
HPA3	AKKVFKRLEKLFSKIWNWK-NH_2	2449.2	-0.97	0.68
HPA3NT3	FKRLKKLFKKIWNWK-NH_2	2062.6	-0.6	0.75
